# Lactate as prognostic factor after out-of-hospital cardiac arrest treated by therapeutic hypothermia

**DOI:** 10.1186/2197-425X-3-S1-A200

**Published:** 2015-10-01

**Authors:** JC Orban, M Novain, F Cattet, R Plattier, M Nefzaoui, H Hyvernat, O Raguin, M Kaidomar, C Ichai

**Affiliations:** Nice University Hospital, Medical Surgical ICU, Nice, France; Nice University Hospital, Medical ICU, Nice, France; Antibes General Hospital, Medical Surgical ICU, Antibes, France; Frejus General Hospital, Medical Surgical ICU, Frejus, France

## Introduction

Out-of-hospital cardiac arrest (OHCA) is a major public health problem whose prognosis is uncertain. Therapeutic hypothermia improved outcome of these patients but also changed the value of several prognosis factors [1, 2]. Lactate is a controversial factor associated to outcome after OHCA [3].

## Objectives

Evaluation of lactate as prognosis factor after out-of-hospital cardiac arrest treated by therapeutic hypothermia

## Methods

We performed a retrospective study including adult patients admitted in 4 ICUs for OHCA and treated by therapeutic hypothermia. We collected demographic data, circumstances of cardiac arrest and arterial lactate levels in the first 48 hours after admission. Neurologic outcome was evaluated by the CPC score: favorable outcome (scores 1-2) and unfavorable outcome (scores 3-5). Data are expressed as median and IQR. Comparisons were made by a Mann-Whitney or a chi-squared tests as appropriate. Odds ratio associated to outcome were estimated by a logistic regression. A p value < 0.05 was considered as statistically significant.

## Results

Two-hundred and seventy-two patients were included in the study: 89 favorable and 183 unfavorable outcome patients. Favorable outcome patients were younger (60 [49-70] vs. 68 [58-76] years; p < 0.01), exhibited shorter durations of no-flow (1 [0-5] vs. 5 [0-10] min; p < 0.01) and low-flow (10 [5-15] vs. 20 [15-30] min; p < 0.01), and a higher proportion of shockable rhythm (74% vs. 30%; p < 0.01). Lactate levels at different times were lower in the favorable outcome patients (table 1).

**Table 1:** Lactate levels according to outcome.

	All patients	Favorable outcome	Unfavorable outcome	p values
Lactate H0	4.2 [2.2-8.1]	2.2 [1.5-3.6]	5.4 [3.3-9.4]	< 0.01
Lactate H12	2.1 [1.3-3.4]	1.4 [1.0-2.2]	2.5 [1.6-4.7]	< 0.01
Lactate H24	1.5 [1.1-2.4]	1.3 [0.9-2.1]	1.8 [1.1-2.8]	0.01
Lactate H48	1.4 [1.1-2.1]	1.3 [1.0-1.8]	1.4 [1.1-2.5]	0.05

In multivariate analysis, several factors were associated to unfavorable outcome: admission lactate levels (OR 1.304 [1.146-1.483] p < 0.01), age (OR 1.031 [1.01-4.55] p < 0.01), non-shockable rhythm (OR 1.993 [1.039-3.825] p < 0.01). Outcome was different according to quartiles of lactate (p < 0.01; figure).

**Figure 1:**
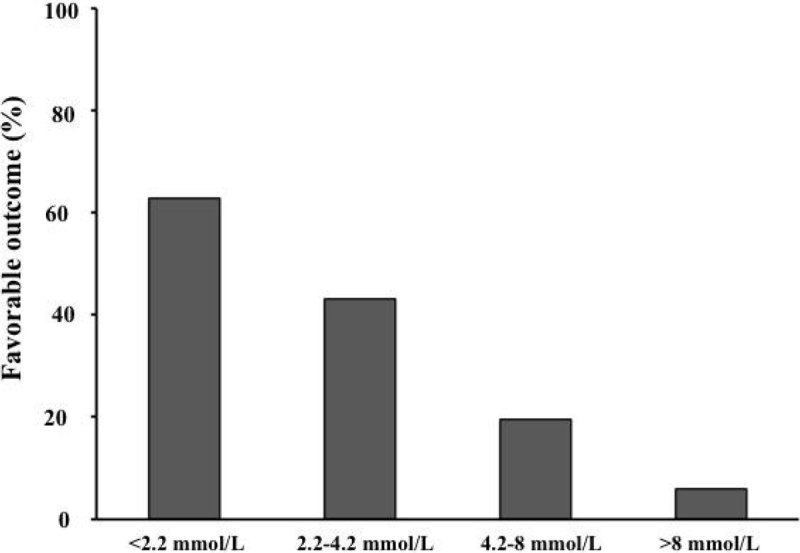
**Outcome according to quartiles of lactate**.

## Conclusions

Lactate levels seem associated to outcome after OHCA treated by therapeutic hypothermia. However, the statistical performance of this test alone is insufficient to establish a neurologic prognosis. Prospective studies associating several parameters are needed to improve prognostication.
